# Cross-sectional relationship of perceived familial protective factors with depressive symptoms in vulnerable youth

**DOI:** 10.1186/s12888-018-1618-x

**Published:** 2018-02-07

**Authors:** Hanna E. Schwendemann, Heidi Kuttler, Thomas Mößle, Eva Maria Bitzer

**Affiliations:** 1 Pädagogische Hochschule Freiburg/University of Education, Kunzenweg 21, 79117 Freiburg, Germany; 2 Cooptima, Talweg 44, 79540 Lörrach, Germany; 3 State Police College Baden-Württemberg, Sturmbühlstraße 250, 78054 Villingen-Schwenningen, Germany

**Keywords:** depressive symptoms, adolescents, familial protective factors, resilience, family structure

## Abstract

**Background:**

There are multiple negative consequences associated with heavy episodic drinking and close associations between substance abuse and depression, alcohol-intoxicated adolescents (AIA) represent a vulnerable group. We aim to add to the current literature by investigating the cross-sectional relationship of perceived familial protective factors with depressive symptoms in AIA in hospitals, with respect to sex. Depression is among the 10 leading causes of disabilities during childhood and adolescence, with girls being more vulnerable than boys. Considerable evidence reveals a strong association between depression and alcohol abuse. The family provides the possibility to positively influence depressive symptoms.

**Methods:**

We present cross-sectional data of a German multisite, epidemiological cohort study on AIA. By using youth’s self-reports, we assessed sociodemographic data, as well as data on perceived familial protective factors and depressive symptoms using items of the Communities that Care Youth Survey instrument. We performed descriptive and multigroup analyses to evaluate the measurement invariance of the used instruments. Moreover, to investigate the relationships between the constructs, we used structural equation modelling.

**Results:**

The study sample comprised 342 AIA, with a mean age of 15.5 years (SD = 1.2; 48.1% girls). The final structural equation model achieved an acceptable model fit of χ^2^ (69, 342) = 110.056; *p* = .001; TLI = 0.97; CFI = 0.98; RMSEA = 0.046; SRMR = 0.042, and the rewards for prosocial involvement in the family context correlated significantly negatively with present depressive symptoms, (ß = − 0.540, *p* <  0.001). The effects were stronger in boys (ß = − 0.576, *p* <  0.001) than in girls (ß = − 0.519, *p* <  0.001).

**Conclusion:**

In vulnerable youth in Germany, depressive symptoms are correlated to good experiences within the family. Future research should assess whether interventions that enhance parental support reduce the risk of depressive symptoms in AIA. Our findings highlight the need for family-based prevention programmes, particularly for AIA with an increased risk of depression.

## Background

Approximately 15–20% of all children and adolescents experience depressive symptoms during childhood and adolescence, and depression is among the 10 leading causes of disability in this age group [1–4]. In Germany, almost 18% of children and adolescents experience such symptoms at least once in their lifetime, and in 4% of German adolescents, depression persists for a minimum of 2 years [2, 5]. Depressive symptoms in youth are a predictor of depression in adulthood [3, 5]. The female sex is an established factor for depression, with the risk in puberty for girls being twice as high as that for boys [6, 7].

Alcohol is one of the most significant risk factors for morbidity and mortality in young people worldwide, and alcohol misuse and episodic heavy drinking is a significant public health concern [8, 9]. More than one-third of 15–16-year olds in Europe have reported excessive alcohol consumption in the last 30 days, and 13% have been intoxicated in the last 30 days [10]. Regular, augmented, or excessive alcohol consumption is often associated with alcohol abuse in later life, as well as with depression during adolescence. Both frequency and extent of alcohol consumption have emerged as significant predictors of higher levels of depression [11–17], and heavy episodic drinking in adolescence can furthermore be an indicator of already existing disorders. Hospitalised intoxicated adolescents are a vulnerable group whose healthy development is endangered. At the same time, the hospitalization of adolescents because of excessive alcohol consumption presents an opportunity to initiate preventive efforts [18].

Models of risk and protective factors have predicted the onset and progression of disorders [19–22]. The Social Development Model (SDM) provides a framework for explaining healthy or problematic development, in which family environment and social interactions emerge as relevant factors influencing youth’s development [19, 23, 24]. It is evident that (1) opportunities for prosocial involvement and interaction with others, (2) the degree of involvement and interaction, (3) the skills to participate, and (4) receiving rewards for prosocial involvement lead to bonding processes within the social environment and act as protective factors [23, 25–27]. Less attachment to parents as well as family conflicts are risk factors for depression [6]. Currently, few studies have investigated the relationship between resilience, protective factors, and problem behaviour or mental disorders in young people [5, 28].

The family, as an instant, lasting, and influential environment for children and youth, protects against the development of psychopathology and provides opportunities for preventive interactions [29–31]. Parents are the central caretakers of children and youth, may influence their attitudes and behaviours, and contribute to their socialisation [32, 33]. Adolescents’ family relationship, family climate, and cohesion can be a risk or a protective factor against depression [5, 34, 35]. Better parent–adolescent communication, parental connectedness, shared activities, and warmth are associated with fewer mental disorders and are strongly protect against depressive symptoms [7, 36].

In this study, we present cross-sectional results of a German multisite, epidemiological cohort study on adolescents hospitalised due to alcohol intoxication (International Classification of Diseases, Tenth Revision (ICD 10) F10.0). Because of the multiple negative consequences associated with heavy episodic drinking and close associations between substance abuse and depression, alcohol-intoxicated adolescents (AIA) represent a vulnerable group. The influence of protective factors in the context of depressive symptoms in AIA has not been well studied. We aim to add to the current literature by investigating the cross-sectional relationship of family protective factors with depressive symptoms in AIA, using the SDM.

In the current study, we examined whether according to the SDM, opportunities for prosocial involvement in the family context lead to rewards for prosocial involvement and cause a good attachment to the mother and father. Furthermore, we investigated whether in compliance with the SDM, good bonding to one’s parents reduces depressive symptoms in AIA. Because depressive symptoms vary with sex, we analysed whether group differences existed in AIA.

## Methods

### Study design

We collected cross-sectional data in a German multisite, epidemiological cohort study, conducted between June 2012 and October 2013. The survey was conducted in co-operation with 10 prevention centres throughout Germany applying the national prevention programme HaLT/“Close to the limit” [9]. This programme targets youth aged 11–17 years hospitalised with acute alcohol intoxication (ICD-10 F10.0) diagnosed by the referring physician in the emergency room. It aims to prevent the stabilisation of heavy alcohol consumption among children and adolescents [9]. HaLT involves two components: a proactive strategy/structural prevention component in the community setting and a reactive strategy, where social workers execute a brief intervention. This intervention is based on motivational interviewing, at patients’ bedside, typically the day after the intoxication incident [9, 37, 38]. To conduct the current survey, the trained social workers recruited the survey participants before the brief intervention and interviewed AIA aged 13–18 years, during their hospital stay.

We defined inclusion criteria (participants should be aged 13–18 years and hospitalised due to alcohol intoxication) and exclusion criteria (not in the desired age group and another reason for hospitalisation). Informed consent of both parents and adolescents was collected by the social workers, who subsequently handed over a written questionnaire to the adolescents. Prevention centres documented all cases of acute alcohol intoxication during the study period and number of study participants. The study participation was 69%. Non-participation was based on either refusal to participate in the survey (15.5%), acute need of support (10.9%), or low literacy (4.6%) [39]. The participating adolescents received a USB flash drive and a 10-Euro voucher as an incentive.

### Measures

Perceived familial protective factors and depressive symptoms were assessed based on self-reports of the youth as well as sociodemographic data (sex, age, and family structure).

#### Familial protective factors

Perceived familial protective factors were recorded using the German version [40] of the Communities That Care (CTC) Youth Survey instrument [19]. The CTC instrument is designed to assess a broad set of risk and protective factors across different domains: community, school, family, peers, and individuals, as well as health and behavioural outcomes [19].

The CTC instrument evolved from the SDM and has been used in the USA and other countries. In Germany, there is an adapted German version of the CTC instrument, which we used in our study [41, 42]. For our analysis, four scales of the CTC-protective family domain with 11 items are included. This selection is based on our previous study on the psychometric properties of the CTC instrument [18]. The attachment of adolescents to their mother and father is measured by three items each. For example, “Do you feel close to your mother?” and “Do you feel close to your father?” Opportunities for prosocial involvement in the family context are measured by three items (e.g., “My parents notice when I am doing a good job and let me know about it”) and rewards for prosocial involvement by two items (e.g., “My parents ask me what I think before most family decisions affecting me are made”). The response categories for the attachment to the mother and father are measured using the following scores: 1 = ‘no’, 2 = ‘rather no’, 3 = ‘rather yes’, and 4 = ‘yes’. Response categories for opportunities and rewards for prosocial involvement ranged from 1 = ‘very wrong’ to 4 = ‘very right’. When summing each scale, high scores represent high familial protection. The Cronbach’s α of the scales are sufficient, attachment to mother: α = 0.80, attachment to father: α = 0.88, opportunities for prosocial involvement: α = 0.74, and rewards for prosocial involvement: α = 0.87 [18].

We followed the recommendation of Arthur et al. (2007) to calculate cut-off values for the scales (whether protective factor is present) [43]. This implies three steps: 1) identifying the scale mean, 2) calculating the mean absolute deviation (MAD) according to Leys (2013) [44], 3) deducting the scale mean with 15*MAD (cut-off points: attachment to mother = 2.85, attachment to father = 2.85, opportunities for prosocial involvement = 3.26, and rewards for prosocial involvement = 3.39) [45].

#### Depressive symptoms

We assessed depressive symptoms with the following items of the CTC instrument [19]: “Sometimes I think life is not worth it,” “All in all, I am inclined to think I am a failure,” and “In the past year, have you felt depressed or sad MOST days, even if you felt okay sometimes?” The response options ranged as follows: 1 = ‘no’, 2 = ‘rather no’, 3 = ‘rather yes’, and 4 = ‘yes’. Cronbach’s α in our AIA sample was 0.80.

To analyse present depressive symptoms, we summed the answers of all three items of the scale. We assumed depressive symptoms, if at least one answer to one of the three items was ‘yes’ or ‘rather yes’, and the sum of the added values has more than 6 points. A recent study on American students (age M = 19.2, SD = 0.44 years) showed a high accuracy of the four-item depression scale of the CTC instrument compared with the Patient Health Questionnaire with 9 items (PHQ-9). The four items reflect the Diagnostic and Statistical Manual of Mental Disorders-5 criteria very well, and three of the four items measure cognitive symptoms, which are strongly discriminant of other depressive symptoms [46].

### Statistical analysis

We calculated frequencies and tested patterns of the missing values. The overall frequency of missing values was low (77.8% of respondents with less than 5% missing data). The missing values were either completely missing at random or missing at random.

Descriptive statistics (mean and proportions) were calculated, and the data were checked for normality. We analysed the construct validity of the familial protective scales and depressive symptoms through confirmatory factor analysis (CFA). As advised by Cole (2007), we allowed correlations between residual terms of the attachment to the mother and father scales, that are implied by the measurement strategy, which are the equally expressed items of the mother and father [47].

We performed structural equation modelling (SEM) to investigate the relationship between the family protective factors and depressive symptoms in AIA using the SDM.

First, we tested the measurement invariance of the SEM multigroup modelling with respect to sex. Configural invariance is present if the factor structure, loading pattern, and intercepts are similar in both groups [48]. For testing weak invariance, the factor loadings are set equal across groups. If this models proves the stage, structural relationship between groups, such as factor correlations, can be examined and compared across groups [49]. Strong invariance is tested by additionally constraining the intercepts to be equal across groups. Confirmation allows a comparison of latent means and regression parameters between groups [50–52]. The nested model is compared with the previous less restricted model by a *χ*^2^ difference test. As noted by Chen (2007), *χ*^2^ differences have the same problem as absolute *χ*^2^ tests by being highly sensitive to the sample size and violations of the normality assumption. Therefore, goodness-of-fit statistics are recommended. When the sample size is adequate (*n* > 300), a change of the Δ comparative fit index (CFI) ≤ − 0.010 supplemented by a change in the Δ root mean square error of approximation (RMSEA) ≤ 0.015 indicates invariance [53]. An analysis of partial measurement invariance is possible, if the nested model is worse than the previous model. Partial invariance is present if at any of the restrictions of the aforementioned stages are freed for some indicators to improve the model fit [48]. If at one stage, partial invariance is present, then this partial model is the basis for the next step of assessing measurement invariance [48].

To analyse the differences in path coefficients with respect to sex, we performed model comparisons by using the *χ*^2^ differences of the restricted model (equal constraint regressions) and the model without the restriction. For model comparisons, we used the Satorra–Bentler-scaled *χ*^2^ difference test using difference test scaling correction and the differences in the degree of freedom [54].

We applied the maximum likelihood estimator with robust standard errors (MLR) to obtain appropriate fit indices [55]. We used the CFI, Tucker–Lewis Index (TLI), and RMSEA to evaluate the model fit. Furthermore, we checked the standardised root mean square residual (SRMR < 0.1). The following parameter estimates and goodness-of-fit statistics describe an acceptable model fit; CFI and TLI ≥ 0.90, RMSEA ≤0.08. Moreover, a good model fit is represented by the following estimates; CFI and TLI ≥ 0.97 and RMSEA ≤0.05, which were used to evaluate the model data [56].

For the descriptive and multivariate analyses, we used SPSS Version 22 (IBM Corp. Released 2013. IBM SPSS Statistics for Windows, Version 22.0. Armonk, NY: IBM Corp). For the CFA, SEM, and measurement invariance tests, we used R (Version 3.2.4) with the missForest, semTools and lavaan packages (0.5–20) [55, 57, 58].

## Results

### Subject characteristics

The study cohort consisted of 342 AIA in hospital, with a mean age of 15.5 years (SD = 1.2, 48.1% girls). Among all adolescents, 46.8% lived in a traditional family. Among the remaining 53.2% of adolescents, most lived with a single mother (Table 1).

**Table 1:** Sociodemographic variables of the study population (*n* = 342)

	N valid	*n* (%)
Female sex	337	162 (48.1)
Traditional family^a^	340	159 (46.8)
Familial protective factors		
ᅟAttachment to mother (good)	321	198 (61.7)
ᅟAttachment to father (good)	316	159 (50.3)
ᅟOpportunities for prosocial involvement (good)	324	173 (53.4)
ᅟRewards for prosocial involvement (good)	323	166 (51.4)
Depressive Symptoms	337	104 (30.9)
ᅟSometimes I think life is not worth it	332	97 (29.2)
ᅟAll in all, I am inclined to think I am a failure	331	72 (21.2)
ᅟIn the past year have you felt depressed or sad MOST days, even if you felt okay sometimes?	331	88 (26.6)
Age	310	M = 15.5 (SD = 1.2)
≤ 13		18 (5.8)
14–15		120 (35.1)
16–17		169 (49.4)
≥ 18		3 (0.9)

*M* mean, *SD* standard deviation

^a^traditional family – living with both parents

### Confirmatory factor analysis

**Fig. 1:**
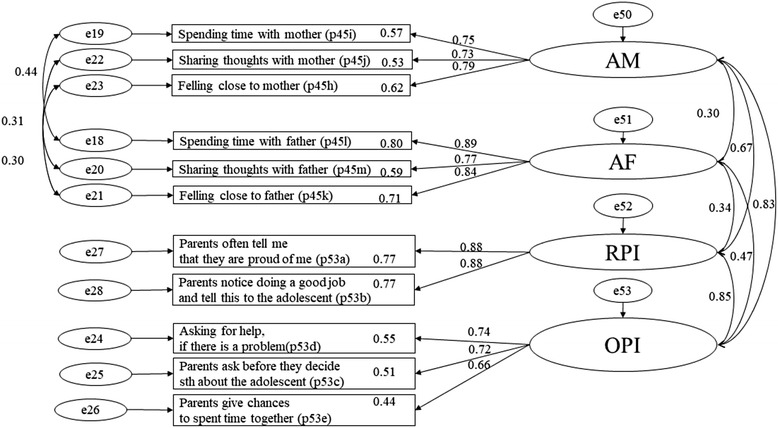
CFA of familial protective factors, AM – attachment to mother / AF – attachment to father/ OPI – opportunities for prosocial involvement / RPI – rewards for prosocial involvement

**Fig. 2:**
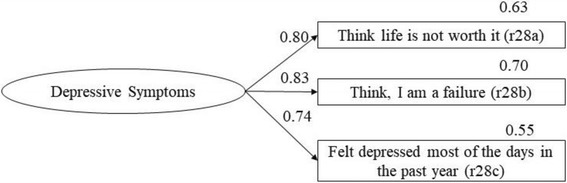
CFA of depressive symptoms

First, we investigated the latent structure of the perceived familial protective factors and depressive symptoms. The four familial protective scales (*χ*^2^ (35; 342) = 56.380, *p* = 0.012; TLI = 0.979; CFI = 0.985; RMSEA = 0.047, SRMR = 0.029) and depressive symptoms represented independent factors. All estimated measurement loadings were significant, and the goodness-of-fit statistics was acceptable (Figs. 1 and 2).

### Relationship between perceived familial protective factors and depressive symptoms

**Fig. 3:**
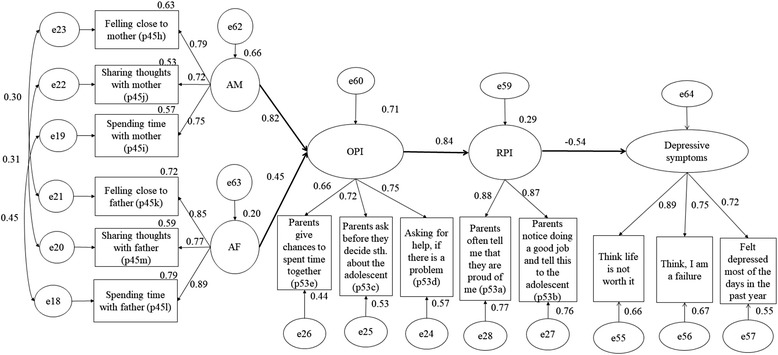
SEM of familial protective factors and depressive symptoms in AIA (n = 342),: AM – attachment to mother / AF – attachment to father / OPI – opportunities for prosocial involvement / RPI – rewards for prosocial involvement

We analysed the relationship of perceived familial protective factors with depressive symptoms using the SDM (*χ*^2^ (69, 342) = 162.847; *p* <  0.001; TLI = 0.94; CFI = 0.95; RMSEA = 0.070; SRMR = 0.065). In the SDM, opportunities for prosocial involvement in the family led to rewards for this involvement. Positive engagement in the family context resulted in a good attachment to the mother and father. We assume, that there is a possibility that the SDM is conceivable in either direction; Therefore, we analysed the SEM vice versa. This led to the model that fitted best to the data. The SDM-reverse model achieved a better model fit of *χ*^2^ (69, 342) = 110.056; *p* = 0.001; TLI = 0.97; CFI = 0.98; RMSEA = 0.046; SRMR = 0.042; Fig. 3).

Because of the cross-sectional data, we analysed the influence of depressive symptoms on perceived rewards for prosocial involvement as well, which resulted in a comparable model adjustment (*χ*^*2*^ (67, 342) = 107.947; *p* = 0.001; TLI = 0.97; CFI = 0.98; RMSEA = 0.047; SRMR = 0.038).

Our cross-sectional analysis revealed that a good attachment to the mother (ß = 0.815, *p* <  0.001) and father (ß = 0.447, *p* <  0.001) led to opportunities for prosocial involvement in the family context. Opportunities for prosocial involvement were significantly positively associated with rewards for prosocial involvement in the family context (ß = 0.844, *p* <  0.001). The SEM showed that rewards for prosocial involvement were significantly negatively related to depressive symptoms. The higher the perceived familial rewards for prosocial involvement were pronounced, the lower was the symptom appearance of depression (ß = − 0.540; *p* <  0.001). Vice versa, we observed that adolescents with higher levels of depressive symptoms perceived lower rewards for prosocial involvement in the family context (ß = − 0.538, *p* < 0.001).

### Measurement invariance

Table 2 shows the results of the hierarchical measurement invariance with respect to sex. The fit statistics of the analysis showed that configural and weak invariance can be assumed. With respect to strong invariance, the changes in the CFI and RMSEA compared with the weak model were larger than − 0.010 and 0.015, respectively. In order to test partial weak invariance, the modification indices for individual parameters were examined. By freeing the loadings of the items p45h (“Feeling close to mother”) and r28a (“Sometimes I think life is not worth it”), the ΔCFI and ΔRMSEA were below their cut-off points, indicating a partial strong invariance.

**Table 2:** Results of the measurement invariance analysis of the SEM comparing boys and girls (MLR estimator)

Model	*χ* ^2^	*df*	Δ *χ*^2^	*p*	CFI	ΔCFI	RMSEA	ΔRMSEA
Thresholds						≤ − 0.010		≤ 0.015
Configural invariance	245.462	134		< 0.001	0.950		0.074	
Weak invariance	254.394	143	8.932	< 0.001	0.950	−0.000	0.072	0.002
Strong invariance	284.859	152	30.465	< 0.001	0.941	−0.009	0.076	−0.002
Partial strong invariance^a^	266.094	151	11.700	< 0.001	0.949	−0.001	0.071	0.001

^a^Freeing of the intercepts of the items p45h and r28a

### Familial protective factors and depressive symptoms with respect to sex

**Fig. 4:**
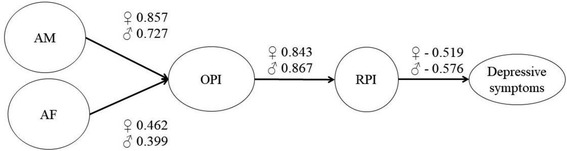
SEM of familial protective factors and depressive symptoms in AIA – differences across sex (n = 342): AM – attachment to mother /AF – attachment to father / OPI – opportunities for prosocial involvement / RPI – rewards for prosocial involvement

We analysed the final SEM model for differences between boys and girls, using multigroup analysis. We tested the nested model, where we constrained the path coefficients of the SEM against the unconstrained model. Sex showed a moderating effect on the SEM Δ *χ*^2^ = 11.335 (Δ df = 4, *p* = 0.023). Compared with girls, boys perceive their mothers and fathers to provide less opportunities for prosocial family involvement (mothers: boys, ß = 0.727, *p* < 0.001 and girls, ß = 0.857, *p* < 0.001; fathers: boys, ß = 0.399, *p* < 0.001 and girls: ß = 0.462, *p* < 0.001). For engagement in the family, boys perceive more rewards (ß = 0.867, *p* < 0.001) than do girls (ß = 0.843, *p* < 0.001). These rewards significantly reduce depressive symptoms in both boys (ß = − 0.576, *p* < 0.001) and girls (ß = − 0.519, *p* < 0.001). A comparison of the latent means between girls and boys in the multigroup analysis revealed that boys were less affected by depressive symptoms (ΔM = − 0.438; Wald-z = − 4.588; *p* < 0.001) and less attached to their mothers (ΔM = − 0.276; Wald-z = − 3.621; *p* < 0.001) than were girls (Fig. 4).

## Discussion

In this study, we analysed cross-sectional data of the vulnerable target population of AIA with respect to perceived familial protective factors and their relationship to depressive symptoms during hospital stay.

One-third of AIA experience depressive symptoms. The prevalence of depressive symptoms in AIA is comparable with that in representative samples [39]. Furthermore, more than 50% of AIA grow in non-traditional families, which accounts for an important risk factor for mental disorders, such as depression [5, 34]. A comparison of perceived familial resources of AIA with those of a representative sample of adolescents clearly showed that AIA have fewer opportunities and rewards for prosocial involvement, but the attachment to parents was almost the same in AIA and representative samples [59, 60].

We proved that the CTC scales used to measure perceived familial protective factors and depressive symptoms represent independent latent factors using CFA. The SEM was partial strong invariant in boys and girls, which is an important prerequisite for meaningful group comparisons.

Our data fit best to a SEM resembling a sort of ‘reversed SDM’. According to the SDM, opportunities and rewards for prosocial engagement are important for fostering familial prosocial socialisation that results in attachment to both parents [26, 61]. Our final model indicates that a good attachment to the mother and father offers opportunities for prosocial involvement in the family context, which results in rewards for prosocial involvement and reduces depressive symptoms. In adolescence, a good relationship to both parents is central for gaining autonomy. Parents with a secure attachment are more responsive and more supportive toward their children to gain autonomy and enable decision-making in adolescents. The search for physical closeness in childhood is replaced with communication about thoughts and feelings of the adolescents [62–64]. The phenomenon of a reverse-SDM path in vulnerable youth should be investigated in a representative sample of adolescents.

As indicated in the literature, adolescents’ family relationship, family climate, and cohesion protect against depression [5, 34, 35]. We showed that in AIA, perceived parental rewards for prosocial involvement as developmental resources are negatively correlated with depressive symptoms. This result helps social workers to support AIA and their family in the situation of hospitalisation as an important starting point for addressing preventive interventions.

Multigroup analysis revealed that these results are valid for both boys and girls. However, significant differences existed between boys and girls. Girls perceive having more opportunities to engage in the family context obtained from their mothers, but boys perceive getting more rewards for prosocial involvement. These rewards result in a significantly stronger reduction of depressive symptoms in boys. With respect to mean differences in depressive symptoms in AIA, we observed that girls had more depressive symptoms than did boys. Studies have reported considerable sex differences not only in the number of depressive symptoms but also in the structure of protective factors. For instance, spending time with the family is particularly protective for boys buffering depressive symptoms [65, 66].

The fact that 53.2% of AIA live with only one parent as well as the negative influence of familial protective factors on depressive symptoms highlights the importance of integrating mothers and fathers within the context of AIA. Furthermore, when working with AIA having depressive symptoms, focus should be placed on familial protective factors. Parental support and emotional warmth confer protective against depressive symptoms [35, 67]. Increasing familial protective factors predicts a decrease in depressive disorders [5]. The incidence rate of depression is considerably higher in adolescents with low parental support than in their high parental support counterparts [1]. AIA have less familial protective factors than representative samples, are more likely to live with a single parent, and have a higher risk of depressive symptoms. Therefore, in AIA, it is necessary to thoroughly evaluate depressive symptoms, family structure, familial protective factors, and sex to prevent a problematic development. One possibility for developing familial protective resources is the ‘Strengthening Families Programme’, which addresses parents and their vulnerable youth to improve familial communication and interaction, clarifying rules and emphasising caring and warm relationship [68, 69].

The protective function of family factors is particularly important for single parents because the risk factors for mental disorders and substance abuse in adolescence accumulate when there are, for example, family conflicts, financial problems, and lack of joint activities [70]. This risk is especially present in boys [71]. Addressing family protective factors and integrating the second parent, which is the father in most cases, can be an important resource in the family environment of adolescents. Familial protective factors are correlated with depressive symptoms in AIA; therefore, the opportunity to address these factors in the context of hospitalisation should be used.

### Strengths and limitations

One of the strengths of this study is the multicentre design integrating vulnerable youth during their hospital stay. It is difficult to reach AIA in routine care, but our study consulted in collaboration with social workers examined more than 300 AIA at 10 hospitals in Germany. We assessed depressive symptoms in hospitalised AIA and perceived familial protective factors; our results add to the scarce literature on this vulnerable target group. We assessed familial protective factors via self-reports because of the survey setting; the feasibility of this empirical inquiry has been indicated in the literature [5, 34, 36, 72]. To confirm the assessment of depression, we cross-checked AIA self-reported depression with the estimated need to initiate social support (i.e., contacting the youth welfare office, drug and family counselling) collected from trained social workers. AIA reporting depressive symptoms received more offers of social support than did the youth not reporting depressive symptoms [39, 45].

Our results are limited by the fact that we only assessed depressive symptoms with three items of the CTC instrument and not via a standardised instrument, such as the PHQ-9, the Beck Depression Inventory, or a diagnostic interview [46]. In this study, we used the CTC instrument to obtain an impression of depressive symptoms in AIA. Briefly, we did not want to diagnose depression, but rather to identify the need for action in adolescents and provide this information to health care professionals outside the hospital setting. It must be considered that we asked the youth for depressive symptoms in the year before hospitalisation. In our analyses, we could only evaluate the relationship of perceived familial protective factors with reported depressive symptoms at the hospital because of the cross-sectional nature of the data. Data on the mid-term development (a 6-month follow-up) of depressive symptoms are available, but were not included in the present study because its small sample size did not allow for a valid analysis. Furthermore, this decision was taken due to differing time spans in the assessment of depressive symptoms. At the hospital, items were used with the original wording, and at T1, the time span was changed for the 6 months since hospitalisation [39].

In addition to familial influences on depressive symptoms, peers play an important role in the lives of adolescents. Their influence can limit the influence of parents [7, 73]. This idea may be integrated in further research on factors protecting against depressive symptoms in AIA, but was not included in the present analysis.

Future research should evaluate the medium-term effects of protective factors on the development of depressive symptoms in AIA. The emergency setting could be used for assessing the risk profiles of AIA that focus on developmental hazards, as well as individual and familial protective factors to identify at-risk groups and offer opportunities for interventions. An intervention study would provide a good opportunity to investigate the medium-term effects of interventions in enhancing familial protective factors in AIA.

## Conclusion

The present findings reveal relationships among depressive symptoms in girls and boys and good experiences within the family, a good attachment to the mother and father, opportunities for prosocial involvement, and rewards for this positive involvement in the family context. The cross-sectional buffering effect of rewards for prosocial involvement is particularly strong in boys. Identifying depressive symptoms in hospitals can be an important starting point for preventive programmes and early interventions to reduce the existing symptoms by reinforcing familial resources; additionally, familial protective factors are helpful for addressing the needs of adolescents at a risk of major depression.

Future research should investigate whether interventions that enhance parental support reduce the risk of depressive symptoms in adolescents after alcohol intoxication. Strengthening familial protective factors may influence the onset of symptoms, and existing symptoms may also be affected by familial resources, such as family climate and social support [5]. Our findings highlight the need for family-based prevention programmes, particularly for AIA having an increased risk of depression.

## Abbreviations

AIA: Alcohol intoxicated adolescents
CFA: Confirmatory factor analysis
CFI: Comparative fit index
CTC: Communities that care youth survey instrument
MAD: Mean absolute deviation
MLR: Maximum likelihood estimator with robust standard errors
PHQ-9: Patient health questionnaire with 9 items
RMSEA: Root mean square error of approximation
SDM: Social development model
SEM: Structural equation model
SRMR: Standardised root mean square residual
TLI: Tucker–Lewis Index
